# Acute kidney injury in burn patients admitted to the intensive care unit: a systematic review and meta-analysis

**DOI:** 10.1186/s13054-019-2710-4

**Published:** 2020-01-02

**Authors:** Torgeir Folkestad, Kjetil Gundro Brurberg, Kine Marie Nordhuus, Christine Kooy Tveiten, Anne Berit Guttormsen, Ingrid Os, Sigrid Beitland

**Affiliations:** 1 Department of Anaesthesiology and Intensive Care Medicine, Haukeland University Hospital, Bergen, Norway; 2 Centre for Evidence Based Practice, Western Norway University of Applied Sciences, Bergen, Norway; 3 Division for Health Services, Norwegian Institute of Public Health, Oslo, Norway; 4 Faculty of Medicine, University of Oslo, Oslo, Norway; 5 Department of Clinical Medicine, Faculty of Medicine, University of Bergen, Bergen, Norway; 6 Renal Research Group Ullevål, Institute of Clinical Medicine, Faculty of Medicine, University of Oslo, Oslo, Norway; 7 Division of Medicine, Department of Nephrology, Oslo University Hospital, Oslo, Norway; 8 Division of Emergencies and Critical Care, Department of Anaesthesiology, Oslo University Hospital, Oslo, Norway

**Keywords:** Acute kidney injury, Burn, Critical illness, Risk factor, Mortality, Renal replacement therapy, Outcome, Mortality, Systematic review

## Abstract

**Background:**

Acute kidney injury (AKI) is a common complication in burn patients admitted to the intensive care unit (ICU) associated with increased morbidity and mortality. Our primary aim was to review incidence, risk factors, and outcomes of AKI in burn patients admitted to the ICU. Secondary aims were to review the use of renal replacement therapy (RRT) and impact on health care costs.

**Methods:**

We conducted a systematic search in PubMed, UpToDate, and NICE through 3 December 2018. All reviews in Cochrane Database of Systematic Reviews except protocols were added to the PubMed search. We searched for studies on AKI according to Risk, Injury, Failure, Loss of kidney function, and End-stage kidney disease (RIFLE); Acute Kidney Injury Network (AKIN); and/or Kidney Disease: Improving Global Outcomes (KDIGO) criteria in burn patients admitted to the ICU. We collected data on AKI incidence, risk factors, use of RRT, renal recovery, length of stay (LOS), mortality, and health care costs.

**Results:**

We included 33 observational studies comprising 8200 patients. Overall study quality, scored according to the Newcastle-Ottawa scale, was moderate. Random effect model meta-analysis revealed that the incidence of AKI among burn patients in the ICU was 38 (30–46) %. Patients with AKI were almost evenly distributed in the mild, moderate, and severe AKI subgroups. RRT was used in 12 (8–16) % of all patients. Risk factors for AKI were high age, chronic hypertension, diabetes mellitus, high Total Body Surface Area percent burnt, high Abbreviated Burn Severity Index score, inhalation injury, rhabdomyolysis, surgery, high Acute Physiology and Chronic Health Evaluation II score, high Sequential Organ Failure Assessment score, sepsis, and mechanical ventilation. AKI patients had 8.6 (4.0–13.2) days longer ICU LOS and higher mortality than non-AKI patients, OR 11.3 (7.3–17.4). Few studies reported renal recovery, and no study reported health care costs.

**Conclusions:**

AKI occurred in 38% of burn patients admitted to the ICU, and 12% of all patients received RRT. Presence of AKI was associated with increased LOS and mortality.

**Trial registration:**

PROSPERO (CRD42017060420)

## Background

Acute kidney injury (AKI) is a common complication in burn patients admitted to the intensive care unit (ICU), but incidence rates depend upon the burn population studied and AKI definition used [1, 2]. Consensus definitions of AKI are developed to include all severities of AKI and allow comparison between studies; these are the Risk, Injury, Failure, Loss of kidney function, and End-stage kidney disease (RIFLE) [3]; Acute Kidney Injury Network (AKIN) [4]; and Kidney Disease: Improving Global Outcomes (KDIGO) criteria [5].

Several risk factors for AKI are identified in burn patients such as high age, burn injury extent and/or mechanism, and presence of multiple organ failure and/or sepsis [2]. However, the results of prophylactic strategies have so far mostly been disappointing [6]. AKI is a heterogeneous condition ranging from subclinical decline in kidney function to need of renal replacement therapy (RRT). Despite development of international treatment guidelines [5], the practical handling of AKI, and use of RRT, varies substantially across the world [7].

AKI in burn patients is associated with increased mortality [2, 8] and probably also increased length of stay (LOS) [2]. From other patient groups, it has become evident that survivors of AKI are prone to developing chronic kidney disease (CKD) and have increased long-term morbidity and mortality [9]. AKI may also be a burden to the health care system, leading to substantially increased treatment costs, especially related to use of RRT [10].

The purpose of the present study was to review incidence, risk factors, and outcomes of AKI in burn patients admitted to the ICU. Secondary aims were to review the use of renal replacement therapy (RRT) and impact on health care costs.

## Methods

### Study registration

This systematic review and meta-analysis was registered in the PROSPERO database on 12 May 2017 (CRD42017060420) [11]. We report results according to the PRISMA guidelines (Additional file 1).

### Data sources and search strategy

We searched papers published between 1 January 2004 and 3 December 2018 in PubMed, UpToDate, and National Institute for Health and Care Excellence (NICE). All reviews in Cochrane Database of Systematic Reviews except protocols were added to the PubMed search. Searches in PubMed consisted of Medical Subject Headings and text words including acute kidney injury and burn. We searched for ongoing systematic reviews in PROSPERO and conducted hand searches of reference lists.

The search focused on the study population, irrespective of reported intervention, comparison, and outcome. Inclusion was limited to studies of burn patients admitted to an ICU, reporting on AKI as defined by full or modified RIFLE, AKIN, and/or KDIGO criteria. Only publications in English or Scandinavian languages were considered (Additional file 2).

### Study selection

Two collaborators (KMN and CKT) independently screened studies for eligibility according to pre-defined study selection criteria (Additional file 3). Titles, abstracts, and keywords were examined, and full texts were obtained for all potentially relevant records. Studies on trauma patients without burns were excluded as findings are presented elsewhere [12]. Empirical studies comparing AKI and non-AKI patients were included; case reports excluded. Any disagreement was resolved through discussion with a senior author (SB).

### Data extraction

Two independent collaborators (TF and SB) extracted data in duplicate according to a pre-defined data extraction form (Additional file 4). In cases where data points were missing or ambiguously reported, the first and last author of the study were contacted by e-mail up to two times to obtain data. For each study, we extracted detailed information about study sampling, i.e. if the patients were recruited consecutively from an intensive care unit or if the study sample was more narrowly defined.

We extracted data on days to AKI, criteria used, incidence rates, and severity including use of RRT. Many risk factors were assessed, including body mass index (BMI), mean arterial pressure (MAP), Total Body Surface Area (TBSA) percent burnt [13], Abbreviated Burn Severity Index (ABSI) [14], Simplified Acute Physiology Score (SAPS) [15], Acute Physiology and Chronic Health Evaluation (APACHE) [16] score, and Sequential Organ Function Assessment (SOFA) score [17] (Additional file 5). Collected outcome data were renal recovery, ICU and hospital LOS, and mortality.

### Assessment of study quality

Two authors (TF and SB) independently assessed the risk of bias of each included study using the Newcastle-Ottawa quality assessment scale [18].

### Quantitative data synthesis

Meta-analyses and forest plots were prepared in R [19] using the meta [20] and the forest plot [21] packages. We used random effect models with the DerSimonian-Laird estimator since we expected some heterogeneity between studies. Continuous and dichotomous risk factors and outcomes were compared in patients with and without AKI by calculating mean differences (MD) and odds ratios (OR), respectively. Data primarily reported as medians with interquartile ranges were re-expressed into means and standard deviations (SDs) as suggested in the Cochrane handbook [22]. Studies reporting distribution of data only as ranges were excluded from the meta-analyses.

Meta-analyses of proportions were performed on arcsine-transformed data. In an attempt to limit in-between study heterogeneity, it was decided post hoc that meta-analyses of proportions should be confined to studies applying consecutive or random data sampling methods. In contrast, all studies were included in meta-analyses based on the use of control groups.

Risk factors potentially associated with development of AKI were explored in pooled analyses if reported in three or more studies. We generated a forest plot containing summary estimates for multiple risk factors. For dichotomous risk factors, ORs were calculated using the meta package in R. Continuous risk factors were expressed as standardised mean differences (SMDs) using the meta package in R and transformed to OR according to the formula suggested in the Cochrane handbook [23].

### Subgroup analyses

We analysed subgroups on mild (RIFLE R, AKIN 1, KDIGO 1), moderate (RIFLE I, AKIN 2, KDIGO 2), and severe (RIFLE F, AKIN 3, KDIGO 3) AKI, and use of RRT.

### Evaluation of heterogeneity

Statistical heterogeneity among studies was assessed with Cochran’s *Q* test [22] and quantified by the *I*^2^ statistic describing the proportion of total variation due to heterogeneity rather than chance [24, 25].

## Results

### Study selection

**Fig. 1:**
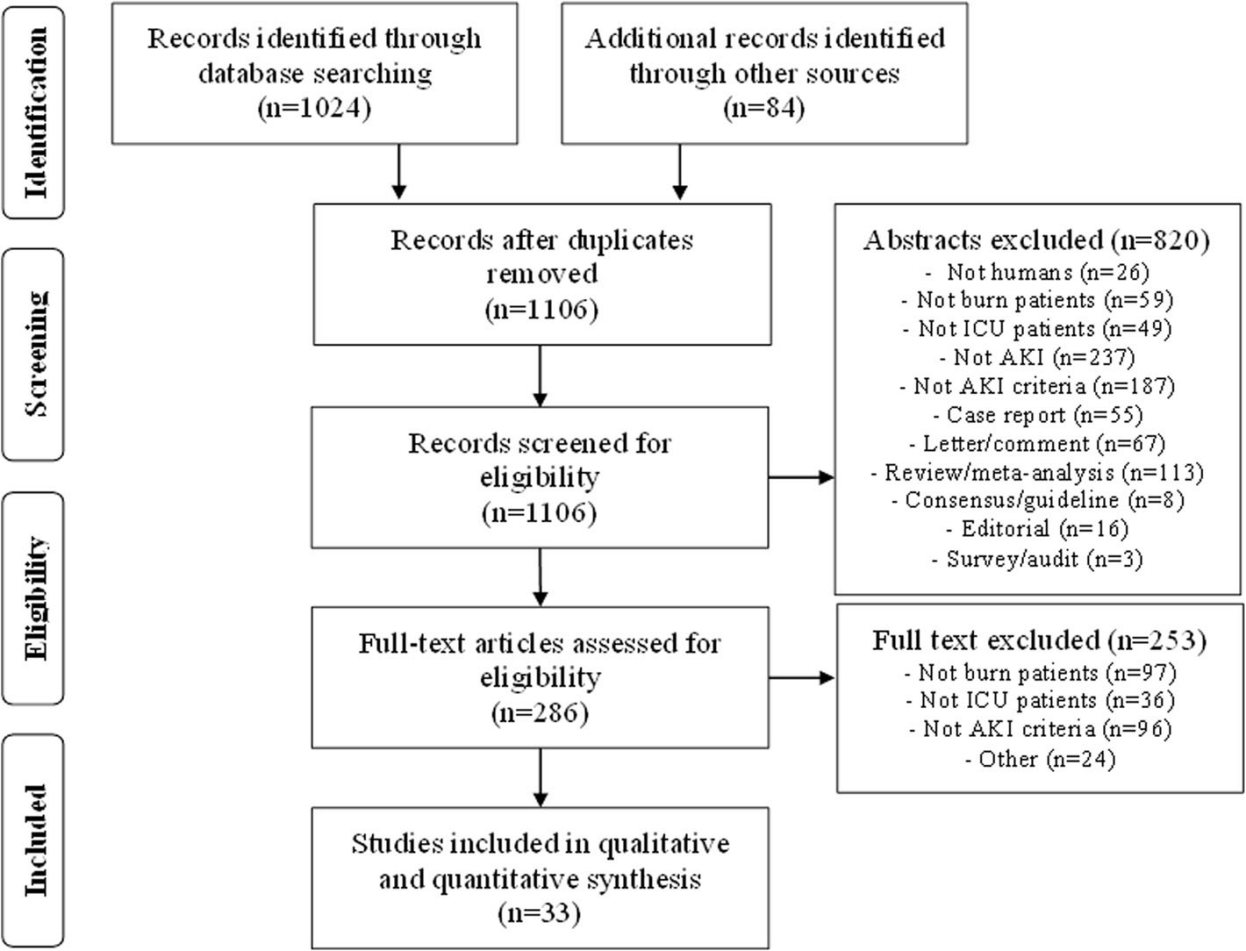
Flow chart of search results according to the Preferred Reporting Items for Systematic reviews and Meta-Analysis (PRISMA) guidelines. ICU, intensive care unit; AKI, acute kidney injury

We identified 1106 unique studies from the literature search and screened their abstracts. Thirty-three of 286 potentially eligible studies were included in the qualitative and quantitative data synthesis [26–58] (Fig. 1). We requested additional data from the authors of nine publications, whereof four provided data [47, 55, 56, 58], one did not have the data [36], and four did not respond [35, 51, 52, 54].

### Study characteristics

All 33 included studies were observational with cohort design published in medical journals with English language in article or letter form. Most studies were on adults with variable burn mechanism and extent. AKI criteria were RIFLE, AKIN, and KDIGO in 18, seven and eight studies, respectively. Eleven studies used original AKI criteria, whereas the remaining used different versions of modified criteria (*n* = 20), or did not describe the use of criteria (*n* = 2) (Table 1).

**Table 1:** Characteristics of included studies on acute kidney injury in burn patients

First author, publication year	Population studied^a^	Consecutive sampling	AKI criteria	Criteria adherence	Study design	Follow-up time AKI^b^	*N*, AKI/total
Lopes JA, 2007	Adults, severe	No	RIFLE	Original	PCS	10 days	n.a.
Coca SG, 2007	Adults, > 10%	Yes	RIFLE	Modified^c^	RCS	Hospital	81/304
Steinvall I, 2008	Adults, > 20%	Yes	RIFLE	Original	PCS	Hospital	31/127
Mariano F, 2008	Adults, severe	No	RIFLE	Unknown	PCS	19 days	n.a.
Palimeri T, 2009	Children, > 10%	Yes	RIFLE	Modified^d^	RCS	Hospital	56/123
Palimeri T, 2010	Adults, > 20%	Yes	RIFLE	Original	RCS	Intensive	32/60
Mosier MJ, 2010	Adults, > 20%	No	RIFLE	Modified^e^	RCS	24 h	n.a.
Schneider DF, 2012	Adults, > 20%	No	RIFLE	Modified^e^	RCS	48 h	n.a.
Chung KK, 2012	Adults, unknown	Yes	AKIN	Modified^c^	RCS	Hospital	656/1973
Hu JY, 2012	Adults, > 30%	Yes	RIFLE	Original	RCS	Hospital	151/396
Stewart IJ, 2013	Adults, burn ICU	No	AKIN	Modified^c^	RCS	Hospital	n.a.
Hong DY, 2013	Adults, > 20%	Yes	RIFLE	Original	PCS	Hospital	11/45
Yang HT, 2014	Adults, > 20%	Yes	RIFLE	Modified^e^	PCS	5 days	31/66
Yavuz S, 2014	Children, > 10%	No	RIFLE	Original	PCS	48 h	n.a.
Noshad H, 2014	Adults, unknown	No	RIFLE	Unknown	PCS	Unknown	n.a.
Howell E, 2015	Adults, > 20%	No	RIFLE	Modified^e^	PCS	48 h	n.a.
Sen S, 2015	Adults, > 20%	No	RIFLE	Modified^f^	PCS	7 days	n.a.
Ren H, 2015	Adults,> 10%	No	KDIGO	Modified^e,g^	PCS	48 h	n.a.
Liang I, 2015	Adults, > 40%	No	RIFLE	Modified^e^	PCS	2 days	n.a.
Yim H, 2015	Adults, major	Yes	AKIN	Original	PCS	28 days	40/97
Kym D, 2015	Adults, > 20%	Yes	RIFLE	Original	PCS	Intensive	48/85
Queiroz LF, 2016	Adults, burn ICU	Yes	KDIGO	Modified^c^	RCS	Intensive	77/293
Rakkolainen I, 2016	Adults, > 15%	Yes	AKIN	Modified^c^	PCS	Intensive	9/19
Sanches-Sanches M, 2016	Adults, > 15%	No	AKIN	Original	PCS	Intensive	n.a.
Kuo G, 2016	Adults, severe	Yes	KDIGO	Modified^e^	RCS	3 days	52/145
Hundeshagen G, 2017	Mix, burn centre	Yes	KDIGO	Modified^c^	RCS	7 days	88/718
Kumar AB, 2017	Adults, > 20%	No	AKIN	Modified^c^	RCS	5 days	n.a.
Kimmel LA, 2018	Adults, > 10%	Yes	RIFLE	Modified^c^	RCS	Unknown	60/267
Chun W, 2018	Adults, > 20%	Yes	AKIN	Original	PCS	28 days	32/76
Depret F, 2018	Adults, > 20%	Yes	KDIGO	Original	PCS	Hospital	55/87
Talizin TB, 2018	Adults, > 20%	No	KDIGO	Modified^c^	PCS	7 days	n.a.
Kim HY, 2019	Adults, surgery	No	KDIGO	Modified^c^	RCS	7 days	n.a.
Clark AT, 2019	Adults, burn ICU	Yes	KDIGO	Modified^c^	RCS	Hospital	601/1040

*AKI* acute kidney injury, *N* number, *RIFLE* Risk, Injury, Failure, Loss of kidney function, and End-stage kidney disease, *AKIN* Acute Kidney Injury Network, *KDIGO* Kidney Disease: Improving Global Outcomes, *PCS* prospective cohort study, *RCS* retrospective cohort study, *ICU* intensive care unit, *n.a* not applicable

^a^Numbers are percent burn injury of Total Body Surface Area

^b^Numbers are minimal follow-up time for AKI

^c^Study used only creatinine criteria and not urine output criteria

^d^Study used paediatric version of criteria

^e^Study used shorter follow-up time than the criteria

^f^Study only used injury and failure according to RIFLE criteria

^g^Study only used serum creatinine increase ≥ 26.5 μmol/L within 48 h

The included studies comprised data from 8200 patients, and 18 of the studies had consecutive sampling of patients. In six of the papers, we selected only patients who had comparison between AKI and non-AKI (Table 1). Most studies reported mean or median age between 30 and 60 years. Male participants ranged from 54 to 100%, and average TBSA percent burnt ranged from 16 to above 70%.

### Assessment of study quality

Overall study quality, scored according to the Newcastle-Ottawa scale, was moderate. The study population consisted of unselected major burn patients in 25 studies, and all studies had comparable control groups. Eleven studies excluded patients with CKD, and 20 studies omitted patients on chronic RRT. Twenty-three studies controlled for confounding factors when comparing groups. Eight of the studies had too short, or undescribed, follow-up time for AKI to occur. Assessment of outcomes was overall satisfactory, but only one study explicitly reported loss to follow-up (Additional file 6). No studies were excluded from our quantitative synthesis due to high risk of bias.

### Quantitative data synthesis

#### Incidence rates

**Fig. 2:**
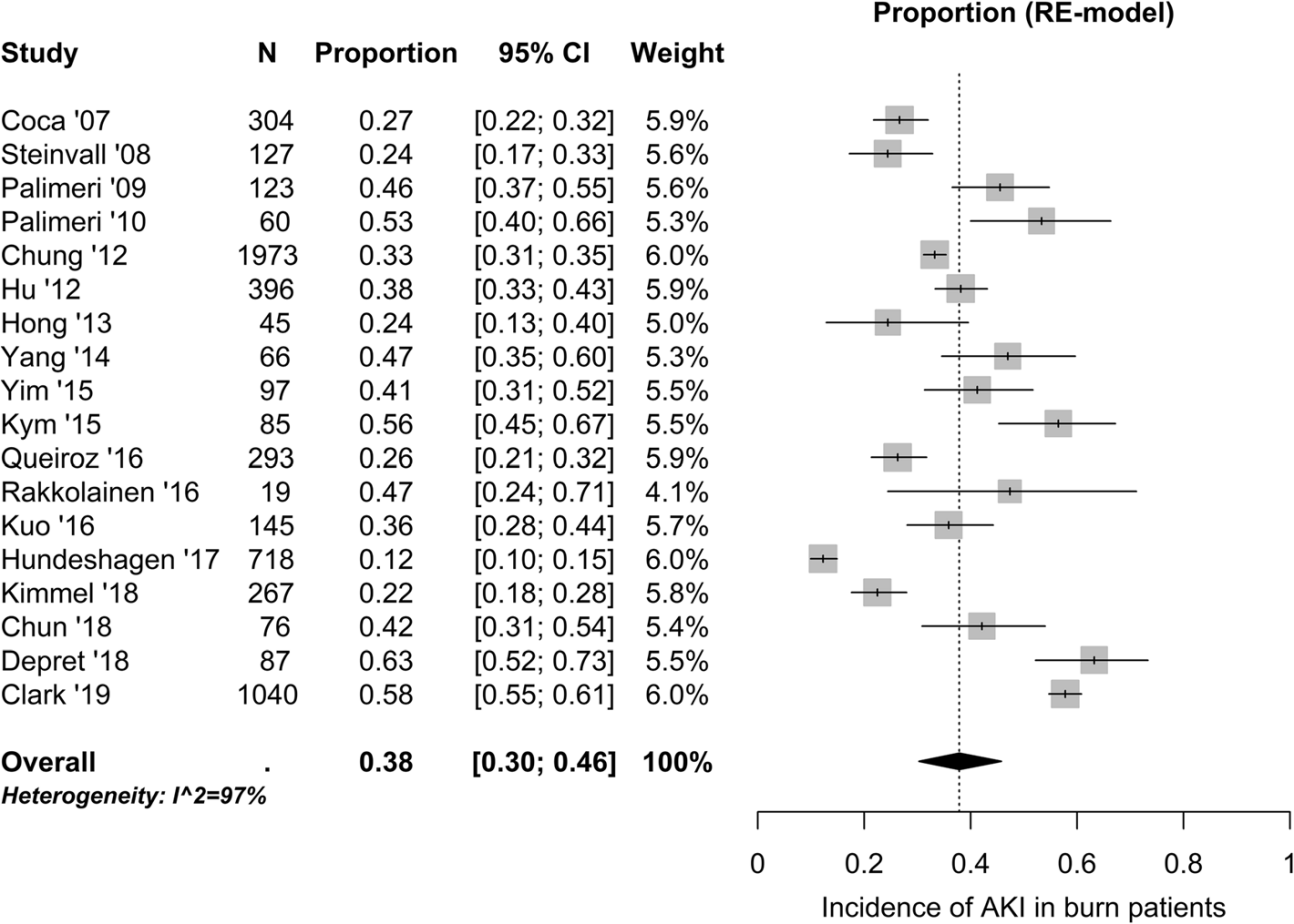
Reported incidence rates of acute kidney injury (AKI) in burn patients admitted to the intensive care unit. *N*, number of patients in the study; CI, confidence interval; RE, random effect

Pooled analysis of 18 studies (5921 patients) with consecutive sampling of patients revealed an overall incidence of AKI of 38 (30–46) % (Fig. 2). Time from burn injury to AKI diagnosis ranged from 1 to 17 days [36, 37, 45, 46, 53]. In the 13 studies reporting incidence rates by AKI severity, 10 (4–18) %, 8 (6–11) %, and 13 (10–17) % had mild, moderate, and severe AKI, respectively (Additional files 7, 8, and 9).

#### Risk factors

**Fig. 3:**
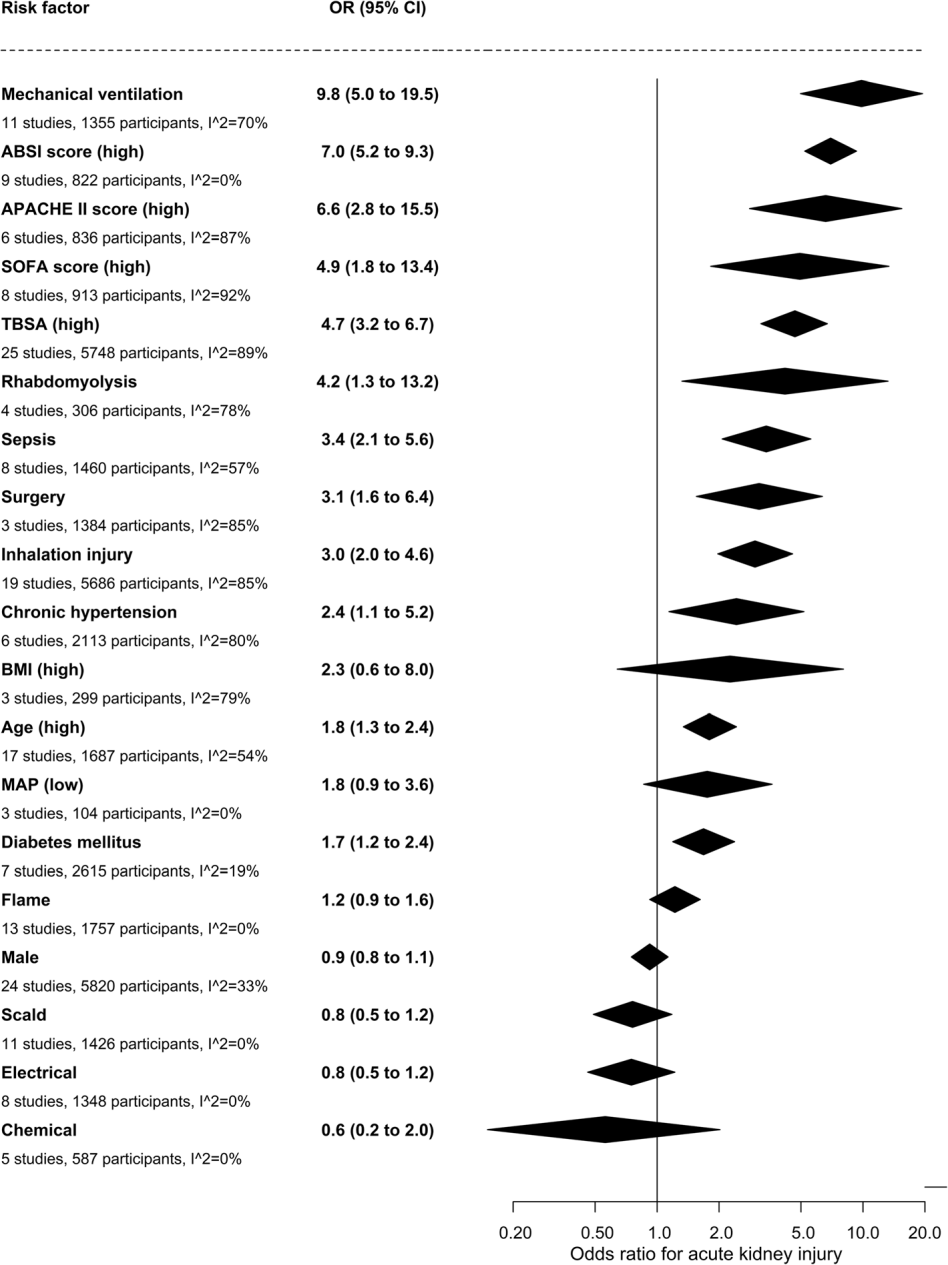
Risk factors for acute kidney injury in burn patients admitted to the intensive care unit. The contribution from the various risk factors were statistically weighted and adjusted to a single scale. Odds ratios (OR) for continuous risk factors were derived from standardised mean differences. CI, confidence interval; ABSI, Abbreviated Burn Severity Index; APACHE, Acute Physiology and Chronic Health Evaluation; SOFA, Sequential Organ Function Assessment; TBSA, Total Body Surface Area; BMI, body mass index; MAP, mean arterial pressure

Risk factors for AKI were reported in 29 studies with 7229 patients (Additional file 5), and pooled analyses yielded crude effect estimates for the different risk factors. High age, chronic hypertension, diabetes mellitus, high TBSA percent burnt, high ABSI score, inhalation injury, rhabdomyolysis, surgery, high APACHE II score, high SOFA score, sepsis, and mechanical ventilation were associated with increased risk of AKI (Fig. 3).

We were unable to quantify the impact of several relevant risk factors because they were reported in fewer than three studies; these included African American descent, body weight, pre-existing coronary artery disease, congestive heart failure and liver failure, SAPS II score, intraabdominal hypertension, circulatory shock, hypotension, number and duration of surgical procedures, and escharotomy (Additional file 5). Additional risk factors could not be analysed because studies reported zero events in both groups; these were pre-existing kidney disease, abdominal compartment syndrome, and chemical injury. Studies reporting median age were excluded because the conversion of median values to means tended to overestimate the risk association. Use of mechanical ventilation and ventilator time were correlated, and we report the use of mechanical ventilation.

#### Renal replacement therapy

RRT was reported in 13 studies (4357 patients) with consecutive sampling of patients and used in 12 (8–16) % of all burn patients (Additional file 10). RRT modes were continuous RRT [38, 45, 46, 54, 55], intermittent haemodialysis [47], or unspecified [27, 28, 34, 37, 48, 50, 58].

#### Length of stay

Nine studies (3069 patients) reported ICU LOS, and 13 studies (4694 patients) hospital LOS. Patients with AKI had 8.6 (4.0–13.2) days longer ICU LOS (Additional file 11) and 10.5 (4.8–16.3) days longer hospital LOS (Additional file 12), compared to non-AKI patients.

#### Mortality

**Fig. 4:**
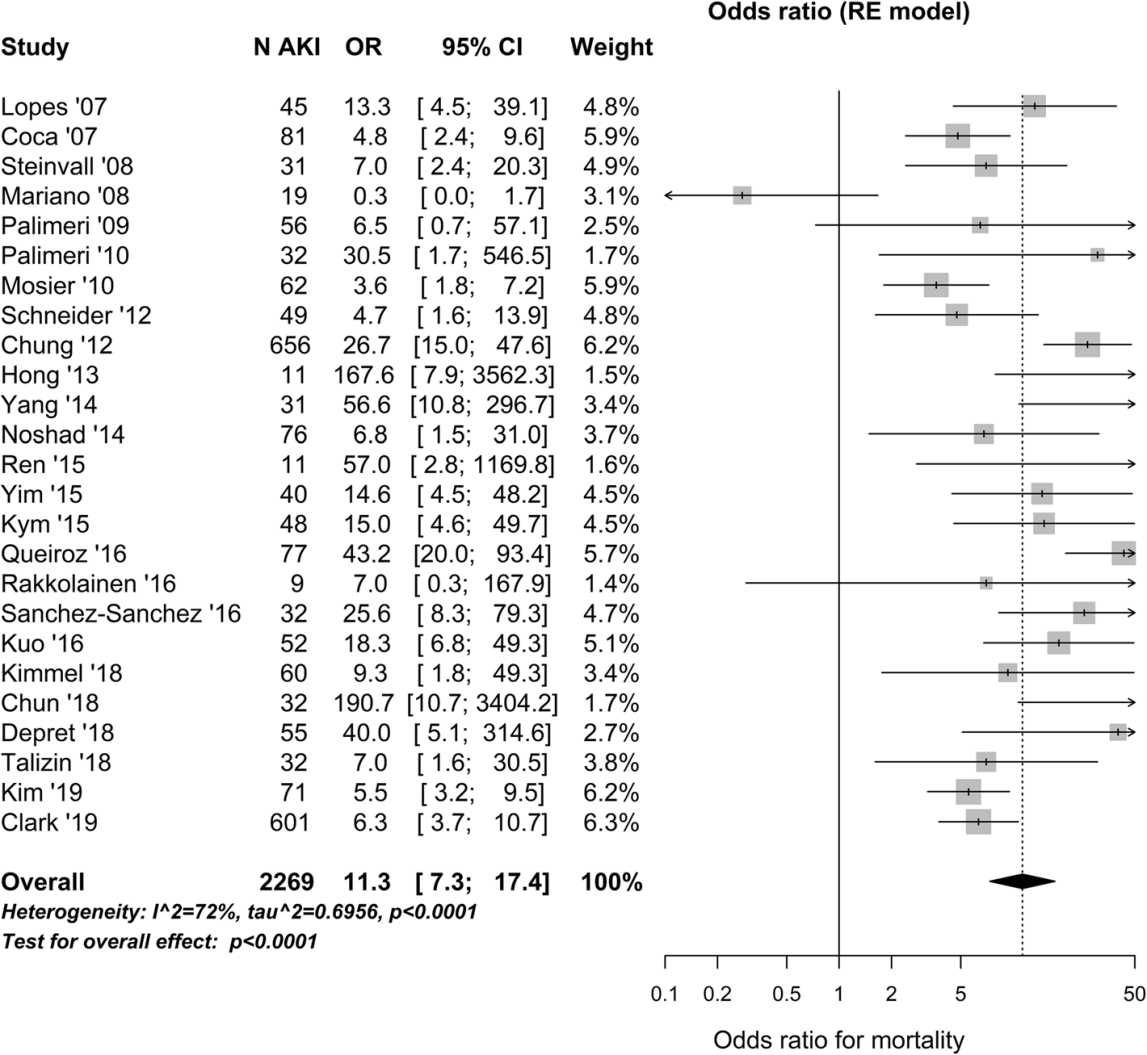
Mortality in burn patients with acute kidney injury (AKI) in the intensive care unit. Odds ratio (OR) for mortality reported at any time point is compared in AKI and non-AKI patients. N AKI, number of AKI patients; CI, confidence interval; RE, random effect

Pooled analysis of 16 studies (1872 AKI patients) revealed that mortality in AKI patients was 43 (32–56) %, but varied considerably across studies (Additional file 13). Mortality was much higher in AKI compared to non-AKI patients, with an OR of 11.3 (7.3–17.4) (Fig. 4).

#### Renal recovery

Renal recovery was reported in two studies (42 AKI patients) with consecutive sampling of patients (Additional file 14), and all patients except two had normal kidney function at discharge [28, 37].

#### Health care costs

None of the studies reported health care costs of AKI.

#### Subgroup analyses

Seven studies (886 AKI patients) reported mortality in the different AKI severity groups. Pooled mortality in mild, moderate, and severe AKI was 14 (7–24) %, 21 (8–38) %, and 67 (51–81) % (Additional files 15, 16, and 17), respectively. AKI compared to non-AKI patients had OR for death of 3.9 (2.0–7.5), 11.1 (5.6–21.6), and 43.0 (23.5–78.8) in mild, moderate, and severe AKI, respectively (Additional files 18, 19, and 20).

Five studies (175 RRT patients) reported that patients undergoing RRT had a mortality rate of 74 (58–87) % (Additional file 21). Six studies (200 RRT patients) revealed that RRT patients had OR for mortality 60.4 (20.1–181.5) compared to non-AKI patients (Additional file 22).

#### Sensitivity analyses

It was decided post hoc that meta-analyses of proportions should be confined to studies that applied consecutive or random data sampling methods, and we therefore performed sensitivity analyses in which all studies were included. Briefly, the results remain similar even though all studies were included. For example, the incidence of AKI remained 38% and mortality among AKI patients remained 43% when all studies were included in the meta-analysis.

#### Heterogeneity

Heterogeneity varied considerably between the meta-analyses. Extensive heterogeneity with Cochran’s *Q* test *p* < 0.0001 and Higgins’ *I*^2^ > 90% was observed in most meta-analyses of proportions. Cochran’s *Q* test also indicated heterogeneity in most analyses of rates and differences, but usually with lower Higgins’ *I*^2^ scores. *I*^2^ was 72% when comparing mortality between AKI and no AKI groups and 77% for the analysis of ICU LOS. It is likely that a large part the observed heterogeneity can be attributed to differences between the available study samples. For example, we show that the mortality increases with the severity of AKI, but the distribution of AKI severity is unknown in many studies. The presence and absence of other risk factors also vary considerably between the included studies, but these differences are difficult to control for without access to individual patient data.

## Discussion

This systematic review reveals that AKI occurs in approximately 38% of burn patients admitted to the ICU, with use of RRT in 12% of all patients. Burn patients at risk for AKI have high age, chronic hypertension, diabetes mellitus, high TBSA percent burnt, high ABSI score, inhalation injury, rhabdomyolysis, surgery, high APACHE II score, high SOFA score, sepsis, and mechanical ventilation. Development of AKI after burn is associated with prolonged stay in ICU and hospital, and reduced chance of survival. Kidney function seems to recover well in most burn patients with AKI. Notably, no study reported the economic consequences of AKI after burns.

A previous study of mixed ICU patients observed that 57% of the patients experienced AKI according to the KDIGO criteria, and 13.5% underwent RRT [59]. In a meta-analysis of burn patients assessed by the RIFLE criteria, AKI was present in 30–66% of the patients, and RRT used in 5% [8]. In comparison, this systematic review using several criteria revealed 38% with AKI and 12% treated with RRT. The incidence of AKI and use of RRT varied widely among the included studies; this may partly be explained by large differences in burn populations. It is likely, however, that many of the studies in this systematic review underreported the incidence of AKI due to the use of modified AKI criteria.

High age, chronic hypertension, and diabetes mellitus are known risk factors for AKI [12, 59]. An earlier meta-analysis found that inhalation injury, high TBSA percent burnt, and high ABSI score were risk factors for AKI after burn [2]. Our data suggest that rhabdomyolysis and surgery are additional burn-related risk factors. AKI is often present in the most severely ill patients as indicated by high APACHE II and SOFA scores [2, 12]. Sepsis and use of mechanical ventilation have also previously been associated with increased risk of AKI in critically ill patients [2, 60, 61].

AKI in ICU patients is often part of multiple organ failure [1, 62, 63]. In line with this, we observed that patients with AKI had more than one week longer ICU and hospital LOS compared to non-AKI patients. A similar observation was recently observed in a meta-analysis of major trauma patients [12]. The effect on LOS in our systematic review may be underestimated, since patients with AKI might have a high early mortality not adjusted for in many of the included studies.

In the present study, AKI after burns was associated with several-fold increased mortality that was worsened with the severity of AKI disease. A previous systematic review of burn patients with AKI according to the RIFLE criteria reported a mortality rate of 35% [8]. When applying several AKI criteria, we found that 43% of burn patients with AKI died, and 74% of patients undergoing RRT. In comparison, mortality was 27% in a study of general ICU patients with AKI [59].

Evaluation of renal recovery is challenging because the definition may vary from full recovery of functional reserve to RRT independence [64]. In our systematic review, only two studies with consecutive sampling of patients reported renal recovery; these reported that all patients except two had normal kidney function at discharge. This finding should be interpreted with caution due to limited number of patients and insufficient follow-up time to evaluate long-term effects. Previous research suggests that ICU patients with AKI have increased risk of CKD and all-cause mortality compared to patients without AKI [9].

None of the studies reported the economic consequences of AKI after burns. Despite this, one would assume that both prolonged LOS and use of RRT would increase treatment costs [65].

This systematic review has a number of clinical limitations. The included studies had large clinical heterogeneity because the study participations and outcome variables varied widely. AKI incidence may be underestimated since many studies used modified AKI criteria. Creatinine levels and urine output are influenced by fluid and/or diuretic therapy not reported in most of the studies. Data on hospital and ICU outcomes are influenced by the local policy for transfer of patients, withholding or withdrawing therapy. The handling of AKI, and particularly the use of RRT, probably varied across sites [66]. Finally, the applicability of the results on renal recovery may be impaired by variable case definitions and short follow-up times.

Methodological limitations are that some publications may have been missed due to language limitation of the literature search. Complete datasets could not be obtained from five studies. Many of our meta-analyses are characterised by substantial statistical heterogeneity, and hence, many summary estimates are uncertain with wide confidence intervals. This heterogeneity is probably caused by heterogeneity in study populations and study design. We have carried out a large number of subgroup analyses aiming to explore what causes the heterogeneity, but it was impossible to single out factors of particular importance. It seems likely that many factors play a role and that the uncertainty would be reduced if we were able to control for confounding variables and present adjusted summary estimates. Unfortunately, this was not possible without access to individual patient data. We did not formally evaluate potential bias that may be caused by use of means and SDs for skewed variables in our analyses of risk factors. Finally, we were unable to include data on economic costs because of missing data.

Strengths of this systematic review are the high number of included studies and patients. Further, the literature search, study selection, and data extraction were determined and published before study start. Two independent collaborators in duplicate screened studies for eligibility, evaluated quality, and extracted data according to pre-set criteria. Finally, we contacted authors twice by e-mail in order to retrieve complete data from eligible publications.

An implication of this systematic review for clinical practice is that health care personnel should be aware of burn patients at risk for AKI, for instance elderly patients with chronic hypertension, diabetes mellitus, and extensive burn injuries. Future studies should explore long-term patient outcomes and treatment costs of AKI among burn victims. There is a clear need for development of uniform standards of reporting in AKI, especially a consensus definition of renal recovery [64, 67].

## Conclusions

The present systematic review reveals that AKI and use of RRT is common in ICU patients with burn injuries. Patients with high age, chronic hypertension, diabetes mellitus, high TBSA percent burnt, high ABSI score, inhalation injury, rhabdomyolysis, surgery, high APACHE II score, high SOFA score, sepsis, and need for mechanical ventilation are at risk for post-burn AKI. Development of AKI after burn has a negative impact on short-time morbidity and mortality, but we lack data on long-term patient outcomes and economic consequences. Limited data suggests that most survivors of AKI regain their kidney function.

## Supplementary information

**Additional file 1.** PRISMA Checklist for systematic reviews Checklist for systematic reviews applied on this manuscript according to the Preferred Reporting Items for Systematic reviews and Meta-Analysis (PRISMA) guidelines.

**Additional file 2.** Literature search strategy. Description of the literature search strategy used in this systematic review.

**Additional file 3.** Study selection form. Description of the study selection process used in this systematic review.

**Additional file 4.** Data extraction form. Description of the data extraction process used in this systematic review.

**Additional file 5.** Overview of reported risk factors for acute kidney injury. Table showing risk factors for acute kidney injury reported in the studies.

**Additional file 6.** Quality assessment of included studies. Table showing the quality assessment of studies according to the Newcastle – Ottawa quality assessment scale.

**Additional file 7.** Incidence of mild acute kidney injury. Figure showing incidence of mild acute kidney injury (AKI). N: Number of patients in the study, CI: confidence interval, RE: random effect.

**Additional file 8.** Incidence of moderate acute kidney injury. Figure showing incidence of moderate acute kidney injury (AKI). N: Number of patients in the study, CI: confidence interval, RE: random effect.

**Additional file 9.** Incidence of severe acute kidney injury. Figure showing incidence of severe acute kidney injury (AKI). N: Number of patients in the study, CI: confidence interval, RE: random effect.

**Additional file 10.** Incidence of renal replacement therapy. Figure showing incidence of renal replacement therapy. N: Number of patients in the study, CI: confidence interval, RE: random effect.

**Additional file 11.** Mean difference in intensive care unit length of stay. Figure showing mean difference in intensive care unit (ICU) length of stay (LOS). N AKI: Number of patients with acute kidney injury (AKI), CI: confidence interval, RE: random effect.

**Additional file 12.** Mean difference in hospital length of stay. Figure showing mean difference in hospital length of stay (LOS). N AKI: Number of patients with acute kidney injury (AKI), CI: confidence interval, RE: random effect.

**Additional file 13.** Mortality in patients with acute kidney injury. Figure showing absolute mortality in patients with acute kidney injury (AKI). N: Number of patients with AKI, CI: confidence interval, RE: random effect.

**Additional file 14.** Incidence of renal recovery. Figure showing incidence of renal recovery. N: Number of patients with acute kidney injury (AKI), CI: confidence interval, RE: random effect.

**Additional file 15.** Mortality in patients with mild acute kidney injury. Figure showing the mortality of patients with mild acute kidney injury (AKI). N: Number of patients with AKI, CI: confidence interval, RE: random effect.

**Additional file 16.** Mortality in patients with moderate acute kidney injury. Figure showing the mortality of patients with moderate acute kidney injury (AKI). N: Number of patients with AKI, CI: confidence interval, RE: random effect.

**Additional file 17.** Mortality in patients with severe acute kidney injury. Figure showing the mortality of patients with severe acute kidney injury (AKI). N: Number of patients with AKI, CI: confidence interval, RE: random effect.

**Additional file 18.** Odds ratio for mortality in patients with mild acute kidney injury. Figure showing the odds ratio (OR) for mortality in patients with mild acute kidney injury (AKI) compared to patients without AKI. N: Number of patients with AKI, CI: confidence interval, RE: random effect.

**Additional file 19.** Odds ratio for mortality in patients with moderate acute kidney injury. Figure showing the odds ratio (OR) for mortality in patients with moderate acute kidney injury (AKI) compared to patients without AKI. N: Number of patients with AKI, CI: confidence interval, RE: random effect.

**Additional file 20.** Odds ratio for mortality in patients with severe acute kidney injury. Figure showing the odds ratio (OR) for mortality in patients with severe acute kidney injury (AKI) compared to patients without AKI. N: Number of patients with AKI, CI: confidence interval, RE: random effect.

**Additional file 21.** Absolute mortality in patients with renal replacement therapy. Figure showing absolute mortality of patients undergoing renal replacement therapy. N: Number of patients on renal replacement therapy, CI: confidence interval, RE: random effect.

**Additional file 22.** Odds ratio for mortality in patients undergoing renal replacement therapy. Figure showing odds ratio (OR) for mortality in patients undergoing renal replacement therapy (RRT) compared to patients without acute kidney injury. N RRT: Number of patients on RRT, CI: confidence interval, RE: random effect.

## Data Availability

The datasets generated and analysed during the current study are available from the corresponding author upon a reasonable request.

## Abbreviations

ABSI: Abbreviated Burn Severity Index
AKI: Acute kidney injury
AKIN: Acute Kidney Injury Network
APACHE: Acute Physiology and Chronic Health Evaluation
BMI: Body mass index
CI: Confidence interval
CKD: Chronic kidney disease
ICU: Intensive care unit
KDIGO: Kidney Disease: Improving Global outcomes
LOS: Length of stay
MAP: Mean arterial pressure
MD: Mean difference
*N*: Number
NICE: National Institute for Health and Care Excellence
OR: Odds ratio
PRISMA: Preferred Reporting Items for Systematic reviews and Meta-Analysis
RE: Random effect
RIFLE: Risk, Injury, Failure, Loss of kidney function, and End-stage kidney disease
RRT: Renal replacement therapy
SAPS: Simplified Acute Physiology Score
SD: Standard deviation
SMD: Standardised mean difference
SOFA: Sequential Organ Function Assessment
TBSA: Total Body Surface Area
