# Supplementary material for: Acute kidney injury in burn patients admitted to the intensive care unit: a systematic review and meta-analysis
Source: Crit Care. 2020 Jan 2;24:2. doi: 10.1186/s13054-019-2710-4 (PMC6941386; doi:10.1186/s13054-019-2710-4)
Supplement: Supplementary file 2 — Additional file 2. Literature search strategy. Description of the literature search strategy used in this systematic review. [file 13054_2019_2710_MOESM2_ESM.docx]

**Additional file 2: Literature search strategy**

**PubMed search 1 (until 3 December 2018)**

("Renal Insufficiency"[Majr:NoExp] OR "Acute Kidney Injury"[Majr] OR "Kidney/injuries"[Majr]) AND ("Multiple Trauma"[Mesh] OR "Microtrauma, Physical"[Mesh] OR Traumatology[Mesh] OR "Trauma Severity Indices"[Majr] OR "Wounds and Injuries"[Majr:NoExp] OR "Wounds, Nonpenetrating"[Majr] OR "Wounds, Penetrating"[Majr] OR "Accidents"[Majr:NoExp] OR "Accidents, Traffic"[Majr] OR "Accidental Falls"[Majr] OR "Burns"[Majr] OR "Bites and Stings"[Majr] OR "Cold Injury"[Majr] OR "War-Related Injuries"[Majr] OR "Electric Injuries"[Majr] OR "Self Mutilation"[Majr] OR "Trauma Centers"[Majr] OR "Emergency Service, Hospital"[Majr:NoExp] OR "Trauma, Nervous System"[Majr] OR Violence[Majr] OR "Shock, Traumatic"[Majr] OR "Crush Injuries"[Majr] OR "Amputation, Traumatic"[Majr] OR "Barotrauma"[Majr] OR "Lacerations"[Majr] OR trauma[Title] OR traumatic[Title] OR neurotrauma*[Title] OR multitrauma*[Title] OR polytrauma*[Title] OR accident[Title] OR accidents[Title] OR blunt[Title] OR (multiple[Title] AND (fracture*[Title] OR injury[Title] OR injuries[Title]))) AND (("2004/01/01"[PDAT] : "2018/12/31"[PDAT]) OR "last 14 years"[PDat]) AND (english[lang] OR norwegian[lang] OR swedish[lang] OR danish[lang])

**PubMed search 2 (To retrieve articles not yet entered into Medline, until 3 December 2018)**

(kidney[Title] OR renal[Title] OR aki[Title]) AND (trauma[Title] OR traumatic[Title] OR neurotrauma*[Title] OR multitrauma*[Title] OR polytrauma*[Title] OR accident[Title] OR accidents[Title] OR wound*[Title] OR stab[Title] OR blunt[Title] OR crush[Title] OR crushing[Title] OR shot*[Title] OR shoot*[Title] OR lacerat*[Title] OR burn*[Title] OR (multiple[Title] AND (fracture*[Title] OR injury[Title] OR injuries[Title]))) AND (("2004/01/01"[PDAT] : "2018/12/31"[PDAT]) OR "last 14 years"[PDat]) AND (english[lang] OR norwegian[lang] OR swedish[lang] OR danish[lang]) NOT medline[sb]

**Cochrane Database of Systematic Reviews (until 3 December 2018)**

Searched Cochrane Reviews via PubMed.

Added to PubMed search 1 and 2, see above:

AND "Cochrane Database Syst Rev"[Journal]

**UpToDate (until 3 December 2018)**

Contents > Nephrology and hypertension > Renal failure: Browsing for acute kidney injury

**NICE (UK) (until 3 December 2018)**

[NICE Guidance](https://www.nice.org.uk/guidance) > [Conditions and diseases](https://www.nice.org.uk/guidance/conditions-and-diseases) > [Kidney conditions](https://www.nice.org.uk/guidance/conditions-and-diseases/kidney-conditions) > Acute kidney injury

**Prospero (until 3 December 2018)**

Acute kidney injury

**Additional sources (until 3 December 2018)**

No search strategy
