# Supplementary material for: Acute kidney injury in burn patients admitted to the intensive care unit: a systematic review and meta-analysis
Source: Crit Care. 2020 Jan 2;24:2. doi: 10.1186/s13054-019-2710-4 (PMC6941386; doi:10.1186/s13054-019-2710-4)
Supplement: Supplementary file 3 — Additional file 3. Study selection form. Description of the study selection process used in this systematic review. [file 13054_2019_2710_MOESM3_ESM.docx]

### Additional file 3: Study selection form

**Reviewer, date:_____________________________________________________________________**

**Number, first author, year:_­­_________________________________________________________**

**Review summary:**

**Decision made by one reviewer (after fulfilling this study selection form)**

Include Ο (all questions below answered “yes”)

Discuss Ο (some question below answered “unknown”)

Exclude Ο (some question below answered “no”)

**Detailed review**

**1. Is it an empirical study?**

Yes: Ο No: Ο Unknown: Ο

**2. Is the study reporting data on acute kidney injury (AKI) in burn patients?**

Yes: Ο No: Ο Unknown: Ο

3. Is the population human burn patients admitted to the intensive care unit (ICU)?

Yes: Ο No: Ο Unknown: Ο

**4. Is the intervention acute kidney injury with one of the following definitions: RIFLE, AKIN or**

**KDIGO?**

Yes: Ο No: Ο Unknown: Ο

**5. Is the comparator burn patients without AKI?**

Yes: Ο No: Ο Unknown: Ο

**6. Reason to exclude**

1. Not burn patients Yes: Ο No: Ο Unknown: Ο

2. Not acute kidney injury Yes: Ο No: Ο Unknown: Ο

3. Not ICU patient Yes: Ο No: Ο Unknown: Ο

4. Not RIFLE, AKIN or KDIGO Yes: Ο No: Ο Unknown: Ο

5. Not humans Yes: Ο No: Ο Unknown: Ο

6. Study design: case report Yes: Ο No: Ο Unknown: Ο

7. Study design: letter, comment of note Yes: Ο No: Ο Unknown: Ο

8. Study design: review or meta-analyse Yes: Ο No: Ο Unknown: Ο

9. Study design: consensus or guideline Yes: Ο No: Ο Unknown: Ο

10. Study design: editorial Yes: Ο No: Ο Unknown: Ο

11. Study design: survey or audit Yes: Ο No: Ο Unknown: Ο

12. Study design: quality improvement Yes: Ο No: Ο Unknown: Ο

13. Study design: study methodology Yes: Ο No: Ο Unknown: Ο

14. Study design: personal observation Yes: Ο No: Ο Unknown: Ο

15. Other reason: ___________________ Yes: Ο No: Ο Unknown: Ο

**Final decision made by the reviewers (after discussion with other reviewer(s))**

Include Ο (all questions below answered “yes”)

Exclude Ο (some question below answered “no”), reason number:__________________________

### 
