# Supplementary material for: Acute kidney injury in burn patients admitted to the intensive care unit: a systematic review and meta-analysis
Source: Crit Care. 2020 Jan 2;24:2. doi: 10.1186/s13054-019-2710-4 (PMC6941386; doi:10.1186/s13054-019-2710-4)
Supplement: Supplementary file 4 — Additional file 4. Data extraction form. Description of the data extraction process used in this systematic review. [file 13054_2019_2710_MOESM4_ESM.docx]

## Additional file 4: Data extraction form

## Reviewer (name, date):______________________________________________________________

**Study number, from list:_____________________________________________________________**

## 1. Study identification

Study identifier (first author, year): __________________________________________________

Study location (country, city):_______________________________________________________

**2. Study method**

Study design:_____________________________________________________________________

**3. Study participants**

Number of patients: Total:_________ With AKI: _________ Without AKI:­­­_________

Type of burn injuries:_______________________________________________________________

Data collection period: ______________________________________________________________

Inclusion criteria:___________________________________________________________________

_________________________________________________________________________________

_________________________________________________________________________________

Exclusion criteria: __________________________________________________________________

_________________________________________________________________________________

_________________________________________________________________________________

AKI definition: _____________________________________________________________________

**6. Results**

| **Outcome reported** | **Dichotomous data** | | **Continuous data** | |
| --- | --- | --- | --- | --- |
| **Specify data** | **With AKI**  N sick /  N exposed | **Without AKI**  N sick /  N exposed | **With AKI** Median / IQR  Mean / SD | **Without AKI**  Median / IQR  Mean / SD |
| Time from burn to AKI (days) |  |  |  |  |
| Risk factor: Age (years) |  |  |  |  |
| Risk factor: Male gender (n) |  |  |  |  |
| Risk factor: African American race (n) |  |  |  |  |
| Risk factor: Weight (kg) |  |  |  |  |
| Risk factor: BMI (kg/m2) |  |  |  |  |
| Risk factor: Pre-existing renal disease (n) |  |  |  |  |
| Risk factor: Diabetes mellitus (n) |  |  |  |  |
| Risk factor: Chronic hypertension (n) |  |  |  |  |
| Risk factor: Coronary artery disease (n) |  |  |  |  |
| Risk factor: Congestive heart failure (n) |  |  |  |  |
| Risk factor: Liver disease (n) |  |  |  |  |
| Risk factor: Severity of illness, SOFA score |  |  |  |  |
| Risk factor: Severity of illness, SAPS score |  |  |  |  |
| Risk factor: Severity of illness, APACHE score |  |  |  |  |
| Risk factor: Total TBSA (%) |  |  |  |  |
| Risk factor: Full thickness TBSA (%) |  |  |  |  |
| Risk factor: ABSI  (score) |  |  |  |  |
| Risk factor: Abdominal comp syndrome (n) |  |  |  |  |
| Risk factor: Circulatory shock (n) |  |  |  |  |
| Risk factor: Mean arterial press (mmHg) |  |  |  |  |
| Risk factor: Hypotension (n) |  |  |  |  |
| Risk factor: Sepsis  (n) |  |  |  |  |
| Risk factor: other: |  |  |  |  |

| **Outcome reported** | **Dichotomous data** | | **Continuous data** | |
| --- | --- | --- | --- | --- |
| **Specify data** | **With AKI**  N sick /  N exposed | **Without AKI**  N sick /  N exposed | **With AKI** Median / IQR  Mean / SD | **Without AKI**  Median / IQR  Mean / SD |
| Risk factor: Ventilator treatment (n) |  |  |  |  |
| Risk factor: Ventilator treatment (days) |  |  |  |  |
| Risk factor: Inhalation injury (n) |  |  |  |  |
| Risk factor: Flame  (n) |  |  |  |  |
| Risk factor: Chemical (n) |  |  |  |  |
| Risk factor: Scald  (n) |  |  |  |  |
| Risk factor: Electrical (n) |  |  |  |  |
| Surgical procedures  (n) |  |  |  |  |
| Surgical procedures  (hours) |  |  |  |  |
| Escarotomi  (n) |  |  |  |  |
| Incidence mild AKI (Rifle R, AKIN 1, KDIGO1)(n) |  |  |  |  |
| Incidence moderate AKI (Rifle I, AKIN 2, KDIGO3)(n) |  |  |  |  |
| Incidence severe AKI (Rifle F, AKIN 3, KDIGO3)(n) |  |  |  |  |
| Creatinine  (micromol/L) |  |  |  |  |
| Urea=Carbamid=BUN  (mmol/L) |  |  |  |  |
| Diuresis  (L) |  |  |  |  |
| Renal replacement therapy at all (n) |  |  |  |  |
| Time from burn to RRT (days) |  |  |  |  |
| Duration of replacement therapy (days) |  |  |  |  |
| Dialysis modus IHD  (n) |  |  |  |  |
| Dialysis modus CRRT  (n) |  |  |  |  |
|  |  |  |  |  |
| **Outcome reported** | **Dichotomous data** | | **Continuous data** | |
| **Specify data** | **With AKI**  N sick /  N exposed | **Without AKI**  N sick /  N exposed | **With AKI** Median / IQR  Mean / SD | **Without AKI**  Median / IQR  Mean / SD |
| ICU LOS  (days) |  |  |  |  |
| Hospital LOS  (days) |  |  |  |  |
| ICU mortality  (n) |  |  |  |  |
| Hospital mortality,  (n) |  |  |  |  |
| Other time mortality,  time: (n) |  |  |  |  |
| Other time mortality,  time: (n) |  |  |  |  |
| Mortality mild AKI (Rifle R, AKIN 1, KDIGO1)(n) |  |  |  |  |
| Mortality moderate AKI (Rifle I, AKIN 2, KDIGO3)(n |  |  |  |  |
| Mortality severe AKI (Rifle F, AKIN 3, KDIGO3)(n) |  |  |  |  |
| Renal recovery (n)  definition: |  |  |  |  |
| Health expenses,  Unit: |  |  |  |  |
| Other data (specify): |  |  |  |  |
| Other data (specify): |  |  |  |  |
