# Supplementary material for: Acute kidney injury in burn patients admitted to the intensive care unit: a systematic review and meta-analysis
Source: Crit Care. 2020 Jan 2;24:2. doi: 10.1186/s13054-019-2710-4 (PMC6941386; doi:10.1186/s13054-019-2710-4)
Supplement: Supplementary file 5 — Additional file 5. Overview of reported risk factors for acute kidney injury. Table showing risk factors for acute kidney injury reported in the studies. [file 13054_2019_2710_MOESM5_ESM.docx]

| **Additional file 5: Risk factors for AKI reported in the studies, page 1** | | | | | | | | | |
| --- | --- | --- | --- | --- | --- | --- | --- | --- | --- |
| **First**  **author** | **Publi-cation year** | **Age** | **Gender** | **African American descent** | **Weight** | **BMI** | **Pre-existing kidney disease** | **Pre-existing diabetes mellitus** | **Pre-existing hyper-tension** |
| Lopes JA | 2007 |  |  |  |  |  |  |  |  |
| Coca SG | 2007 | X | X | X |  |  |  | X |  |
| Steinvall I | 2008 | X | X |  |  |  | X |  | X |
| Mariano F | 2008 | X | X |  |  |  |  |  |  |
| Palimeri T | 2009 | X | X |  |  |  |  |  |  |
| Palimeri T | 2010 | X | X |  |  |  |  |  |  |
| Mosier MJ | 2010 | X | X |  |  | X | X | X | X |
| Schneider DF | 2012 | X | X |  |  |  |  |  |  |
| Chung KK | 2012 | X | X |  |  |  | X |  |  |
| Hu JY | 2012 |  |  |  |  |  | X |  |  |
| Stewart IJ | 2013 |  |  |  |  |  |  |  |  |
| Hong DY | 2013 | X | X |  |  |  |  |  |  |
| Yang HT | 2014 | X | X |  |  |  |  |  |  |
| Yavuz S | 2014 | X | X |  | X |  | X |  |  |
| Noshad H | 2014 |  |  |  |  |  | X |  |  |
| Howell E | 2015 | X | X |  |  |  |  |  |  |
| Sen S | 2015 | X |  |  |  |  | X |  |  |
| Ren H | 2015 | X | X |  |  |  |  |  |  |
| Liang I | 2015 | X | X |  |  | X |  |  |  |
| Yim H | 2015 | X | X |  |  |  | X |  |  |
| Kym D | 2015 | X | X |  |  |  |  |  |  |
| Queiroz LF | 2016 | X | X |  |  |  |  |  |  |
| Rakkolainen I | 2016 | X | X |  |  | X |  |  |  |
| Sanches-Sanches M | 2016 | X |  |  |  |  | X | X | X |
| Kuo G | 2016 | X | X |  |  |  | X | X |  |
| Hundeshagen G | 2017 |  |  |  |  |  | X |  |  |
| Kumar AB | 2017 |  |  |  |  |  |  |  |  |
| Kimmel LA | 2018 | X | X |  |  |  | X | X |  |
| Chun W | 2018 |  |  |  |  |  |  |  |  |
| Depret F | 2018 | X | X |  |  |  |  |  | X |
| Talizin TB | 2018 | X | X |  |  |  |  |  |  |
| Kim, HY | 2018 | X | X |  |  |  | X | X | X |
| Clark AT | 2019 | X | X | X |  |  |  | X | X |
| **Total number of studies with reported risk factor** | | **26** | **24** | **2** | **1** | **3** | **13** | **7** | **6** |

AKI: acute kidney injury, BMI: body mass index.

| **Additional file 5: Risk factors for AKI reported in the studies, page 2** | | | | | | | | | |
| --- | --- | --- | --- | --- | --- | --- | --- | --- | --- |
| **First**  **author** | **Publi-cation year** | **Pre-existing coronary artery disease** | **Pre-existing congest-ive heart failure** | **Pre-existing liver disease** | **SOFA score** | **SAPS II score** | **APACHE II score** | **TBSA** | **ABSI** |
| Lopes JA | 2007 |  |  |  |  |  |  |  |  |
| Coca SG | 2007 |  | X | X |  |  |  | X |  |
| Steinvall I | 2008 |  |  |  |  |  |  | X |  |
| Mariano F | 2008 |  |  |  |  |  |  | X |  |
| Palimeri T | 2009 |  |  |  |  |  |  | X |  |
| Palimeri T | 2010 |  |  |  | X | X | X | X | X |
| Mosier MJ | 2010 | X | X | X |  |  | X | X |  |
| Schneider DF | 2012 |  |  |  |  |  |  | X |  |
| Chung KK | 2012 |  |  |  |  |  |  | X |  |
| Hu JY | 2012 |  |  |  |  |  |  |  |  |
| Stewart IJ | 2013 |  |  |  |  |  |  |  |  |
| Hong DY | 2013 |  |  |  |  |  |  | X | X |
| Yang HT | 2014 |  |  |  |  |  |  | X |  |
| Yavuz S | 2014 |  |  |  |  |  |  | X | X |
| Noshad H | 2014 |  |  |  |  |  |  |  |  |
| Howell E | 2015 |  |  |  |  |  |  | X |  |
| Sen S | 2015 |  |  |  |  |  |  | X |  |
| Ren H | 2015 |  |  |  |  |  | X | X |  |
| Liang I | 2015 |  |  |  | X |  | X | X |  |
| Yim H | 2015 |  |  |  |  |  |  | X |  |
| Kym D | 2015 |  |  |  | X |  |  | X | X |
| Queiroz LF | 2016 |  |  |  | X |  | X | X | X |
| Rakkolainen I | 2016 |  |  |  | X |  |  | X | X |
| Sanches-Sanches M | 2016 |  |  |  | X |  |  | X | X |
| Kuo G | 2016 |  |  |  | X |  | X | X |  |
| Hundeshagen G | 2017 |  |  |  |  |  |  |  |  |
| Kumar AB | 2017 |  |  |  |  |  |  |  |  |
| Kimmel LA | 2018 |  |  |  |  |  |  |  |  |
| Chun W | 2018 |  |  |  |  |  |  |  |  |
| Depret F | 2018 |  |  |  | X | X |  | X | X |
| Talizin TB | 2018 |  |  |  |  |  |  | X | X |
| Kim, HY | 2018 |  |  |  |  |  |  | X |  |
| Clark AT | 2019 |  |  |  |  |  |  | X |  |
| **Total number of studies with reported risk factor** | | **1** | **2** | **2** | **8** | **2** | **6** | **25** | **9** |

AKI: acute kidney injury, SOFA: Sequential Organ Function Assessment, SAPS: Simplified Acute Physiology Score, APACHE: Acute Physiology And Chronic Health Evaluation, TBSA: Total Body Surface Area, ABSI: Abbreviated Burn Severity Index.

| **Additional file 5: Risk factors for AKI reported in the studies, page 3** | | | | | | | | | |
| --- | --- | --- | --- | --- | --- | --- | --- | --- | --- |
| **First**  **author** | **Publi-cation year** | **Abdo-minal compart-ment syndrome** | **Intra-abdo-minal hyper-tension** | **Circul-atory shock** | **MAP** | **Hypo-tension** | **Sepsis** | **Ventilator number** | **Ventilator time** |
| Lopes JA | 2007 |  |  |  |  |  |  |  |  |
| Coca SG | 2007 |  |  |  |  | X | X | X |  |
| Steinvall I | 2008 |  |  |  |  |  |  | X |  |
| Mariano F | 2008 |  |  |  |  |  | X |  |  |
| Palimeri T | 2009 | X |  |  |  |  | X |  | X |
| Palimeri T | 2010 |  |  |  |  |  | X |  | X |
| Mosier MJ | 2010 | X |  |  |  |  | X |  | X |
| Schneider DF | 2012 |  |  |  |  |  |  |  |  |
| Chung KK | 2012 |  |  |  |  |  |  |  | X |
| Hu JY | 2012 |  |  |  |  |  |  |  |  |
| Stewart IJ | 2013 |  |  |  |  |  |  |  |  |
| Hong DY | 2013 |  |  |  |  |  |  | X |  |
| Yang HT | 2014 |  |  |  |  |  | X | X |  |
| Yavuz S | 2014 |  |  |  |  |  |  |  |  |
| Noshad H | 2014 |  |  |  |  |  |  |  |  |
| Howell E | 2015 |  |  |  | X |  |  |  |  |
| Sen S | 2015 |  |  |  | X |  |  |  |  |
| Ren H | 2015 |  |  |  |  |  |  | X |  |
| Liang I | 2015 |  |  |  | X |  |  |  |  |
| Yim H | 2015 |  |  |  |  |  | X |  |  |
| Kym D | 2015 |  |  |  |  |  |  |  |  |
| Queiroz LF | 2016 |  |  |  |  |  |  | X |  |
| Rakkolainen I | 2016 |  |  |  |  |  |  | X |  |
| Sanches-Sanches M | 2016 |  |  |  |  |  |  | X |  |
| Kuo G | 2016 |  |  | X |  |  |  | X |  |
| Hundeshagen G | 2017 |  |  |  |  |  |  |  |  |
| Kumar AB | 2017 |  |  |  |  |  |  |  |  |
| Kimmel LA | 2018 | X |  |  |  |  |  |  | X |
| Chun W | 2018 |  |  |  |  |  |  |  |  |
| Depret F | 2018 |  |  | X |  |  | X | X |  |
| Talizin TB | 2018 |  | X |  |  |  |  | X |  |
| Kim, HY | 2018 |  |  |  |  |  | X |  |  |
| Clark AT | 2019 |  |  |  |  |  |  |  | X |
| **Total number of studies with reported risk factor** | | **3** | **1** | **2** | **3** | **1** | **9** | **11** | **7** |

AKI: acute kidney injury, MAP: mean arterial pressure

| **Additional file 5: Risk factors for AKI reported in the studies, page 4** | | | | | | | | | | |
| --- | --- | --- | --- | --- | --- | --- | --- | --- | --- | --- |
| **First**  **author** | **Publi-cation year** | **Inha-lation**  **Injury** | **Flame injury** | **Chemi-**  **cal**  **injury** | **Scald injury** | **Electri-cal injury** | **Surgical proce-dures, number** | **Surgical proce-dures, time** | **Escaro-tomy** | **Oher risk**  **factors**  **reported** |
| Lopes JA | 2007 |  |  |  |  |  |  |  |  |  |
| Coca SG | 2007 | X |  |  |  |  |  |  |  |  |
| Steinvall I | 2008 |  |  |  |  |  |  |  |  | X^a^ |
| Mariano F | 2008 |  |  |  |  |  |  |  |  |  |
| Palimeri T | 2009 | X |  |  |  | X | X |  |  | X^b^ |
| Palimeri T | 2010 |  |  |  |  |  |  |  |  |  |
| Mosier MJ | 2010 | X | X |  | X |  | X |  | X | X^c^ |
| Schneider DF | 2012 | X | X |  |  |  |  |  |  | X^d^ |
| Chung KK | 2012 | X |  |  |  |  |  |  |  | X^e^ |
| Hu JY | 2012 |  |  |  |  |  |  |  |  |  |
| Stewart IJ | 2013 |  |  |  |  |  |  |  |  |  |
| Hong DY | 2013 | X | X |  | X |  |  |  |  |  |
| Yang HT | 2014 | X | X | X | X | X |  |  |  | X^f^ |
| Yavuz S | 2014 |  | X |  | X |  |  |  |  |  |
| Noshad H | 2014 |  |  |  |  |  |  |  |  |  |
| Howell E | 2015 |  |  |  |  |  |  |  |  |  |
| Sen S | 2015 |  |  |  |  |  |  |  |  |  |
| Ren H | 2015 | X |  |  |  |  |  |  |  | X^f^ |
| Liang I | 2015 | X | X |  | X |  |  |  |  |  |
| Yim H | 2015 | X | X | X | X | X |  |  |  | X^f^ |
| Kym D | 2015 | X | X | X | X | X |  |  |  | X^g^ |
| Queiroz LF | 2016 | X | X | X | X | X |  |  |  |  |
| Rakkolainen I | 2016 | X | X |  | X |  |  |  | X |  |
| Sanches-Sanches M | 2016 | X | X |  |  | X |  |  |  | X^h^ |
| Kuo G | 2016 | X |  |  |  |  |  |  |  |  |
| Hundeshagen G | 2017 |  |  |  |  |  |  |  |  |  |
| Kumar AB | 2017 |  |  |  |  |  |  |  |  |  |
| Kimmel LA | 2018 | X |  |  |  |  |  |  |  | X^i^ |
| Chun W | 2018 |  |  |  |  |  |  |  |  |  |
| Depret F | 2018 | X |  |  |  |  |  |  |  |  |
| Talizin TB | 2018 |  | X | X | X | X |  |  |  |  |
| Kim, HY | 2018 | X | X |  | X | X |  |  |  |  |
| Clark AT | 2019 | X |  |  |  |  | x | X |  |  |
| **Total number of studies with reported risk factor** | | **19** | **13** | **5** | **11** | **8** | **3** | **1** | **2** | **11** |

AKI: acute kidney injury. ^a^Report on multiple organ failure, potentially nephrotoxic exposures and SIRS (Systemic inflammatory response syndrome). ^b^Report on the use of nephrotoxic drugs, vasoactive drugs and PRISM (Pediatric risk of mortality) score, ^c^Report on presence of ARDS (Adult respiratory distress syndrome), ^d^Report on multiple organ failure,^e^Report on ISS (Injury severity score), ^f^Report on presence of rhabdomyolysis, ^g^Report on presence of myoglobinuria, ^h^Report on the use of vasopressor drugs, ^i^Report on premorbid cardiac condition.
