# Supplementary material for: Acute kidney injury in burn patients admitted to the intensive care unit: a systematic review and meta-analysis
Source: Crit Care. 2020 Jan 2;24:2. doi: 10.1186/s13054-019-2710-4 (PMC6941386; doi:10.1186/s13054-019-2710-4)
Supplement: Supplementary file 6 — Additional file 6. Quality assessment of included studies. Table showing the quality assessment of studies according to the Newcastle – Ottawa quality assessment scale. [file 13054_2019_2710_MOESM6_ESM.docx]

**Additional file 6: Quality assessment of included studies**

| **First author, publication year** | **Repres-entative-**  **ness**  **(a)** | **Selection of non-exposed (b)** | **Ascert-ainment of expo-sure (c)** | **Incident disease (d)** | **Compara-bility**  **(e)** | **Assess-ment of outcome**  **(f)** | **Length of follow-up**  **(g)** | **Adequ-acy of follow-up (h)** |
| --- | --- | --- | --- | --- | --- | --- | --- | --- |
| Lopes JA, 2007 | A | A | A | B | A | A | A | D |
| Coca SG, 2007 | A | A | A | B | A | A | A | D |
| Steinvall I, 2008 | A | A | A | B | C | A | A | D |
| Mariano F, 2008^i^ | C | A | A | B | C | A | A | D |
| Palimeri T, 2009 | C | A | A | B | B | A | A | D |
| Palimeri T, 2010 | A | A | A | B | C | A | A | D |
| Mosier MJ, 2010 | A | A | A | A | A | A | B | D |
| Schneider DF, 2012^i^ | A | A | A | B | C | A | B | D |
| Chung KK, 2012 | B | A | A | A | B | A | A | D |
| Hu JY, 2012^i^ | B | A | A | A | A | A | A | D |
| Stewart IJ, 2013 | A | A | A | B | B | A | A | D |
| Hong DY, 2013 | A | A | A | B | A | A | A | D |
| Yang HT, 2014^i^ | A | A | A | B | A | A | B | D |
| Yavuz S, 2014 | C | A | A | A | C | A | A | D |
| Noshad H, 2014 | A | A | A | A | C | A | B | D |
| Howell E, 2015 | A | A | A | B | B | A | B | D |
| Sen S, 2015 | A | A | A | A | B | A | A | D |
| Ren H, 2015^i^ | A | A | A | B | C | A | B | D |
| Liang I, 2015 | B | A | A | A | C | A | B | D |
| Yim H, 2015 | A | A | A | A | A | A | A | D |
| Kym D, 2015 | A | A | A | B | B | A | A | D |
| Queiroz LF, 2016 | A | A | A | B | A | A | A | D |
| Rakkolainen I, 2016 | A | A | A | B | C | A | A | D |
| Sanches-Sanches M, 2016 | A | A | A | A | A | A | A | D |
| Kuo G, 2016 | A | A | A | B | A | A | B | A |
| Hundeshagen G, 2017 | B | A | A | A | B | A | A | D |
| Kumar AB, 2017^i^ | A | A | A | B | A | A | A | D |
| Kimmel LA, 2018 | A | A | A | B | A | A | A | D |
| Chun W, 2018 | A | A | A | B | B | A | A | D |
| Depret F, 2018 | A | A | A | B | A | A | A | D |
| Talizin TB, 2018 | B | A | A | B | C | A | A | D |
| Kim, HY, 2019 | A | A | A | A | A | A | A | D |
| Clark AT, 2019 | A | A | A | B | A | A | A | D |

(a) A, truly representative; B, somewhat representative; C, selected group; D, no description of the derivation of the cohort (b) A, drawn from same community as the exposed; B, drawn from a different source; C, no description of derivation of non-exposed (c) A, secure record; B, structured interview; C, written self-report; D, no description (d) A, the outcome of interest was not present at start of study; B, the outcome of interest may be present at start of study (e) A, controls for demographics/co-morbidities; B, controls for any additional factor (e.g., age, severity of illness); C, not done (f) A, independent/blind assessment; B, record linkage; C, self-report; D, no description (g) A, long enough for outcomes to occur; B, might not be long enough for outcomes to occur (h) A, complete follow-up; B, subjects lost to follow-up was unlikely to introduce bias; C, follow-up rate 90% or lower; D, no statement ^i^Only selected patients from the original study is included
